# Plant-produced transmission blocking *Plasmodium falciparum* Pfs25 subunit and VLP based vaccine candidates

**DOI:** 10.1186/1475-2875-11-S1-O51

**Published:** 2012-10-15

**Authors:** Konstantin Musivchuk, Vadim Mett, Louis Casta, Christine E Farrance, R Mark Jones, Jessica A Chichester, Jennifer Jaje, Slobodanka D Manceva, Moneim Shamloul, Amy Rhee, Will Roeffen, Robert W Sauerwein, Olga Muratova, Yimin Wu, Patrick Duffy, Vidadi Yusibov

**Affiliations:** 1 Fraunhofer USA Center for Molecular Biotechnology, USA; 2 Radboud University Nijmegen Medical Center, Departments of Medical Microbiology, Nijmegen Center for Molecular Life Sciences, The Netherlands; 3 National Institutes of Health, Laboratory of Malaria Immunology and Vaccinology National Institute of Allergy and Infectious Diseases, USA

## 

Malaria is a serious mosquito-borne disease caused by a protozoan parasite. Vaccines can target different stages of the pathogen’s life cycle. Transmission blocking vaccines target mosquito stages of the parasite life cycle, and will support eradication programs to ease the disease burden at the population level. Pfs25 is a sexual stage protein of *Plasmodium falciparum* which is found on the surface of the parasite zygote as it develops in the mosquito midgut. Antibodies against this protein block zygote development, and as a result block transmission to the next human host. Pfs25 was successfully expressed in our plant-based launch vector system as a fusion to the lichenase carrier molecule and as a fusion to the Alfalfa mosaic virus coat protein (AIMV CP), and in each case was purified to a high level of homogeneity. The resulting Pfs25-lichenase and Pfs25-AIMVCP antigens have undergone extensive biochemical characterization and dose-ranging studies in pre-clinical animal models, where both antigens induced transmission blocking antibodies. These data demonstrate the feasibility of expressing Plasmodium antigens in a plant-based system for the economical production of a transmission-blocking vaccine against malaria.

